# Integrated Transcriptomic and Metabolomic Analysis Reveals Metabolic Heterosis in Hybrid Tea Plants (*Camellia sinensis*)

**DOI:** 10.3390/genes16121457

**Published:** 2025-12-05

**Authors:** Yu Lei, Jihua Duan, Feiyi Huang, Ding Ding, Yankai Kang, Yi Luo, Yingyu Chen, Nianci Xie, Saijun Li

**Affiliations:** 1 Tea Research Institute, Hunan Academy of Agricultural Sciences, National Medium and Small Leaf Tea Plant Germplasm Resource Repository (Changsha), Hunan Provincial Engineering Technology Research Center for Tea Variety and Seedling, Changsha 410125, China; leiyu0727@163.com (Y.L.); hncysdjh@hunaas.cn (J.D.); h.fy157@163.com (F.H.); dingding881103@163.com (D.D.); hncyskyk@hunaas.cn (Y.K.); luoyi197912@hunaas.cn (Y.L.); chenyy2405@163.com (Y.C.); cysxnc@hunaas.cn (N.X.); 2 Yuelushan Laboratory, Changsha 410125, China

**Keywords:** tea heterosis, transcriptome, metabolome, lipids, linoleic acid metabolism

## Abstract

Background: Heterosis (hybrid vigor) is a fundamental phenomenon in plant breeding, but its molecular basis remains poorly understood in perennial crops such as tea (*Camellia sinensis*). This study aimed to elucidate the molecular mechanisms underlying heterosis in tea and its hybrids by performing integrated transcriptomic and metabolomic analyses of F_1_ hybrids derived from two elite cultivars, Fuding Dabaicha (FD) and Baojing Huangjincha 1 (HJC). Methods: Comprehensive RNA sequencing and widely targeted metabolomic profiling were conducted on the parental lines and F_1_ hybrids at the one-bud-one-leaf stage. Primary metabolites (including amino acids, nucleotides, saccharides, and fatty acids) were quantified, and gene expression profiles were obtained. Transcriptomic and metabolomic datasets were integrated using KEGG pathway enrichment and co-expression network analysis to identify coordinated molecular changes underlying heterosis. Results: Metabolomic profiling detected 977 primary metabolites, many of which displayed non-additive accumulation patterns. Notably, linoleic acid derivatives (9(S)-HODE, 13(S)-HODE) and nucleotides (guanosine, uridine) exhibited significant positive mid-parent heterosis. Transcriptomic analysis revealed extensive non-additive gene expression in F_1_ hybrids, and upregulated genes were enriched in fatty acid metabolism, nucleotide biosynthesis, and stress signaling pathways. Integrated analysis demonstrated strong coordination between differential gene expression and metabolite accumulation, especially in linoleic acid metabolism, cutin/suberine biosynthesis, and pyrimidine metabolism. Positive correlations between elevated fatty acid levels and transcript abundance of lipid metabolism genes suggest that the transcriptional remodeling of lipid pathways contributes to heterosis. Conclusions: These findings provide novel insights into tea plant heterosis and identify potential molecular targets for breeding high-quality cultivars.

## 1. Introduction

Tea (*Camellia sinensis*) is among the most widely consumed nonalcoholic beverages worldwide, second only to water in popularity. It encompasses a diverse range of types—green, white, black, oolong, and Pu-erh—each characterized by distinctive sensory profiles and cultural significance [1]. The health-promoting effects of tea are primarily attributed to its complex metabolite composition, exemplified by amino acids such as theanine, which is abundant in fresh tea leaves and contributes to its fresh taste and rich flavors [1,2,3]. These metabolites underlie tea’s beneficial properties (e.g., antioxidant and anti-inflammatory effects), as evidenced by metabolomic studies documenting variations in free amino acids, flavonoids, and caffeine across different cultivars and growing conditions [1,4,5]. This metabolic richness positions tea as a valuable model for agricultural research, with implications for improving both crop quality and nutritional value through targeted breeding strategies [6,7,8]. These efforts are supported by high-throughput analytical tools capable of identifying and characterizing metabolites in response to genetic, agronomic, and environmental factors [1,4,9].

Heterosis (hybrid vigor) is a fundamental biological phenomenon widely exploited in plant breeding to enhance desirable traits, such as yield and quality, in economically important crops, including tea [10,11,12,13]. In tea plants, heterosis is particularly evident in volatile compound production, where F_1_ hybrids consistently outperform their parental lines in terpenoid and green leaf volatile content, as demonstrated by gas chromatography–mass spectrometry (GC-MS) analyses [11]. Similar heterotic advantages are observed in other crops. For example, maize hybrids display robust metabolite profiles associated with increased biomass and yield, and rice hybrids such as Taiyou 871 and Taiyou 398 exhibit heterosis in grain quality traits linked to transcriptomic and metabolomic shifts [14,15]. At the molecular level, heterosis in tea involves non-additive expression of key genes and transcription factors, such as CsDXS, CsMYB1, and CsWRKY40, that regulate volatile biosynthesis and enhance aroma in hybrids [11,12]. Likewise, comparative transcriptomic studies in maize reveal diurnal regulation of metabolites and proteins, with hybrids showing non-additive increases in pathways such as carbon assimilation. These findings support a multi-mechanistic model of heterosis involving both dominance and overdominance [16,17]. Despite these advances, the genetic basis of heterosis in tea remains only partially understood, particularly with respect to metabolic contributions. This gap highlights the need for integrated omics approaches to unravel the complex interplay between genotype and phenotype across diverse plant systems [10,12,18].

Research into the tea genome and metabolome has advanced significantly, revealing that primary metabolites contribute to heterosis via specific biochemical pathways, with parallel observations in cereals such as rice and maize [19,20]. Metabolomic analyses of tea have identified widespread non-additive accumulation of metabolites in hybrid plants. Notably, nucleotides (e.g., inosine, guanosine, adenosine), organic acids (e.g., succinic and adipic acids), and tannins are associated with positive heterosis, whereas catechins often show negative heterosis relative to the maternal cultivars [10,16]. This trend extends to nucleotide metabolism pathways: non-additive accumulation of nucleotides serves as a key marker of hybrid vigor in tea, and similarly, rice hybrids show nucleoside accumulation correlated with improved yield and quality [10,15]. For instance, integrative studies of the rice endosperm metabolome reveal alterations in starch and sucrose metabolism, amino acid metabolism, and fatty acid degradation that may influence heterosis outcomes via gene–metabolite coexpression networks [15,21]. This cross-species perspective underscores the potential of metabolomics to identify candidate metabolites and elucidate pathways that underpin heterosis, thereby bridging gaps between crop improvement and molecular biology [14,22,23].

Building on these foundations, we employed a comparative approach to investigate metabolic heterosis in tea by hybridizing parental varieties, Fuding Dabaicha (FD) and Baojing Huangjincha 1 (HJC) with distinct metabolic profiles. We hypothesized that hybrid vigor in tea arises from coordinated non-additive regulation at the metabolic and transcriptomic levels. To test this, we generated F_1_ progeny through controlled crosses and conducted comprehensive transcriptomic and metabolomic profiling. Specifically, high-throughput RNA-seq and widely targeted metabolomics were employed to characterize gene expression patterns and metabolite accumulation across pathways related to primary metabolism. The goal was to integrate transcriptomic and metabolomic data to reveal how molecular interactions in hybrids contribute to metabolic heterosis, thereby informing future strategies for breeding elite tea cultivars with enhanced quality traits [24].

## 2. Materials and Methods

### 2.1. Plant Materials

To generate the F_1_ population, controlled hand pollination was conducted using the parental lines *C. sinensis* cv. Baojing Huangjincha 1, male parent and *C. sinensis* cv. Fuding Dabaicha, female parent. These are widely used, genetically distinct, and differ markedly in key quality traits such as amino acids, polyphenols, and caffeine. Flowers were emasculated and bagged to prevent contamination from foreign pollen. A total of 48 seedlings were obtained and grown under uniform environmental conditions. To verify hybrid authenticity, 32 EST-SSR markers were screened, and 17 primer pairs with clear polymorphism between parents were selected. Among these, three primer sets showing parental-specific alleles were used for the molecular identification of true hybrids. F_1_ individuals exhibiting alleles from both parents were designated as authentic hybrids. The confirmed F_1_ progeny were further analyzed for genetic diversity and heterosis performance in metabolomic and transcriptomic traits in this study.

F_1_ seeds harvested were sown at the experimental tea plantation of the Hunan Tea Research Institute and cultivated under uniform environmental conditions alongside the clonal parent seedlings. All plants received consistent management practices, including standardized irrigation, fertilization, and pruning regimes. During March to April 2024, one-bud-one-leaf shoots were collected from 10 tea trees for each genotype, respectively, mixed as one biological replicate. The samples were then immediately flash-frozen in liquid nitrogen, and stored at −80 °C for subsequent analysis. A total of 300 g of fresh leaf samples were picked from each tea plant variety for metabolome and transcriptome analysis. 100 g of tea leaves were harvested for each biological replicate; we collected three biological replicates [11,16] from the five cultivars.

To assess variation in nutritional quality, we quantified the free amino acid content across the tea population. Free amino acids in tea were quantified using the ninhydrin colorimetric method under alkaline conditions (pH 8.0). The colored reaction products were measured spectrophotometrically at 570 nm, and concentrations were calculated using calibration curves prepared from amino acid standards. The final free amino acid content was expressed as a percentage of dry weight based on the measured absorbance and sample mass. Based on their total free amino acid levels, we identified three F_1_ offspring with markedly different contents—designated as LP, MP, and HP—containing 2.91%, 5.20%, and 6.50%, respectively. The parental cultivars FD and HJC exhibited free amino acid contents of 4.97% and 3.43%, respectively.

### 2.2. Transcriptome Sequencing Preparation and Data Analysis

Total RNA was extracted from plant samples using CTAB-PBIOZOL and Trizol methods, respectively. Following ethanol precipitation, RNA was dissolved in DEPC-treated water and assessed for quality/quantity using a Qubit fluorometer and Qsep400 bioanalyzer (Thermo Fisher Scientific, Waltham, MA, USA). mRNA libraries were constructed via poly(A) selection using Oligo(dT) magnetic beads. Enriched mRNA was fragmented, reverse-transcribed into strand-specific cDNA using random hexamers and dUTP-based second-strand synthesis, followed by end repair, dA-tailing, and adapter ligation. Libraries (250–350 bp inserts) were PCR-amplified, purified with magnetic beads, and validated using Qubit and Qsep400. Pooled libraries underwent 150 bp paired-end sequencing on an Illumina platform employing fluorescent dNTP-based synthesis-by-sequencing chemistry.

Raw reads were quality-controlled with fastp (v0.23.4), removing adapter-containing reads and those with >10% N bases or >50% low-quality bases (Q ≤ 20) [25]. Clean reads were aligned to a species-specific reference genome (downloaded from public repositories) using HISAT2 (v2.2.1). Transcript assembly and novel gene prediction were performed using StringTie 2.1.6 [26], which applies a network flow algorithm to reconstruct full-length transcripts. The tool enables accurate transcript reconstruction and identification of previously unannotated genes through reference-guided and optional de novo assembly approaches. Gene expression was quantified as FPKM (Fragments Per Kilobase Million) using featureCounts (v2.0.3). Differential expression analysis between groups was conducted with DESeq2 (v1.38.3), applying Benjamini–Hochberg FDR correction (adjusted *p* < 0.05, |log_2_FC| ≥ 1). Differential genes underwent enrichment analysis via hypergeometric testing (KEGG pathways, GO terms).

### 2.3. Metabolite Profiling

Metabolite extraction and profiling were performed following a streamlined workflow. Briefly, biological samples were vacuum freeze-dried using a lyophilizer (Scientz-100F, Scientz Biotechnology Co., Ltd., Ningbo, China), then ground into fine powder at 30 Hz for 1.5 min using a grinder (MM 400, Retsch, Haan, Germany). Approximately 30 mg of powdered sample was weighed (MS105DM electronic balance, Mettler-Toledo Instrument Co., Ltd., Zurich, Switzerland) and extracted with 1500 μL of pre-cooled (−20 °C) 70% methanol containing internal standards (lidocaine, L-2-chlorophenylalanine, 2-chloroaniline, phenoxy-D5-acetic acid and benzoyl-D5-glycine). Samples were vortexed for 30 sec every 30 min (6 repetitions total), followed by centrifugation at 12,000 rpm for 3 min. The supernatant was filtered through a 0.22 μm membrane and transferred to vials for UPLC–MS/MS analysis [27].

Chromatographic separation was conducted using a UPLC-ESI-MS/MS system (ExionLC™ AD, SCIEX, Framingham, MA, USA) coupled to a QTRAP^®^ tandem mass spectrometer (SCIEX). The column used was Agilent SB-C18 (1.8 μm, 2.1 mm × 100 mm, Agilent Technologies, Inc., Santa Clara, CA, USA). Mobile phases consisted of 0.1% formic acid in water (solvent A) and 0.1% formic acid in acetonitrile (solvent B). A gradient elution was applied as follows: 95% A/5% B at 0 min; linear gradient to 5% A/95% B over 9 min; held for 1 min; returned to 95% A/5% B in 1.1 min and held for 2.9 min. Flow rate was 0.35 mL/min, column temperature was 40 °C, and injection volume was 2 μL.

The ESI source was operated at 500 °C with spray voltages of +5500 V (positive mode) and −4500 V (negative mode). Gas settings were 50 psi (GSI), 60 psi (GSII), and 25 psi (CUR), with high collision-activated dissociation (CAD). Data acquisition was conducted in multiple reaction monitoring (MRM) mode using nitrogen as the collision gas. MRM transitions were optimized per metabolite by adjusting declustering potential (DP) and collision energy (CE). Each metabolite was monitored within a defined retention window according to its elution characteristics [27].

The Metabolite identification was performed using a two-step approach. First, to identify compounds, we used our in-house metabolite library, which contains chemical standards and a manually curated compound list based on accurate mass (*m*/*z*, ±25 ppm), retention time and spectral patterns [28]. Second, further metabolites were identified based on accurate mass, isotope pattern and MS/MS spectra against public databases, including HMDB, MoNA, MassBank, METLIN, and NIST. Next, the generated MS1/MS2 pairs were automatically searched in the public databases: HMDB (http://www.hmdb.ca/), MoNA (http://mona.fiehnlab.ucdavis.edu/), and MassBank (http://www.massbank.jp/). The MS/MS spectra similarity score was calculated using the forward dot-product algorithm, which considers both fragments and intensities. The similarity score cutoff was set as 0.5 [29]. The MS/MS spectra match was manually checked to confirm the identifications, which was considered a level 2 identification according to MSI [30].

### 2.4. Differential Analysis and Hierarchical Cluster Analysis

To identify differential metabolites, we used orthogonal partial least squares discriminant analysis (OPLS-DA) and selected variables with a variable importance in projection (VIP) score ≥ 1.0. Model quality was evaluated using R^2^X, R^2^Y, and Q^2^ parameters, with Q^2^ values > 0.9, indicating excellent predictive power. To confirm model robustness and avoid overfitting, we conducted 200-permutation tests. The resulting *p*-values (*p* < 0.05) confirmed that the OPLS-DA models were statistically reliable and not overfitted. The hierarchical cluster analysis (HCA) of both samples and metabolites was visualized as heatmaps accompanied by dendrograms. Pearson correlation coefficients (PCCs) among samples were calculated using the cor function in R and displayed solely as heatmaps. Both HCA and PCC analyses were performed with the R package (4.3.3) ComplexHeatmap. For HCA, normalized metabolite signal intensities (unit variance scaling) were represented using a continuous color gradient.

### 2.5. Definition of Expression Patterns Between F_1_ Hybrids and Parental Lines

The Mid-parent heterosis value (MPV) was calculated as previously described: MPV (%) = (F_1_ − MP)/MP × 100, F_1_ = performance of F_1_ generation, HP = performance of high-value parent, MP = mid-parent average [(Parent1 + Parent2)/2] [16]. For metabolic heterosis analysis, the heterosis values were calculated per metabolite. For the correlation analysis, the Pearson correlation coefficients were calculated utilizing the Cor function in R.

### 2.6. Quantitative PCR Analysis

Total RNA was extracted from tea leaf samples using a commercial kit according to the manufacturer’s instructions, including a genomic DNA removal step. The RNA quality was verified before reverse transcription into cDNA. Quantitative real-time PCR was performed using the SYBR Green dye method on three biological replicates. Target genes were analyzed along with one internal reference gene for normalization. Relative gene expression levels were calculated using the comparative 2^−ΔΔCt^ method. All reactions were conducted with appropriate controls to ensure specificity and accuracy of the amplification.

## 3. Results

### 3.1. Transcriptome Profiles of Five Tea Varieties

Using HJC and FD as parental lines, we established an F_1_ hybrid population. From this F_1_ progeny, three varieties (HP, MP, and LP) exhibiting significant differences in total amino acid content were selected for investigating tea nutritional quality. We then conducted transcriptomics analyses on leaf samples from these five varieties. To gain an overview of the gene expression changes in the various tea varieties, leaves at the shooting stage from FD, HJC, HP, MP and LP, were collected and used for the transcriptome analysis. A cDNA library was constructed using RNA extracted from leaf samples of the five tea varieties. Transcriptome sequencing generated three biological replicates per variety, yielding 918,383,346 raw read pairs. After filtering low-quality reads, adapter sequences, and reads containing ambiguous bases (N), 850,769,896 high-quality clean reads (127.61 Gb) were retained. The average base error rate was 0.01%, with Q20 and Q30 values averaging 98.55% and 95.57%, respectively. The average GC content across all samples was 44.41% (Table 1). De novo transcriptome assembly using StringTie software (2.1.6) identified 54,881 expressed genes (FPKM > 0), comprising 31,666 known genes and 23,215 novel genes.

**Figure 1:**
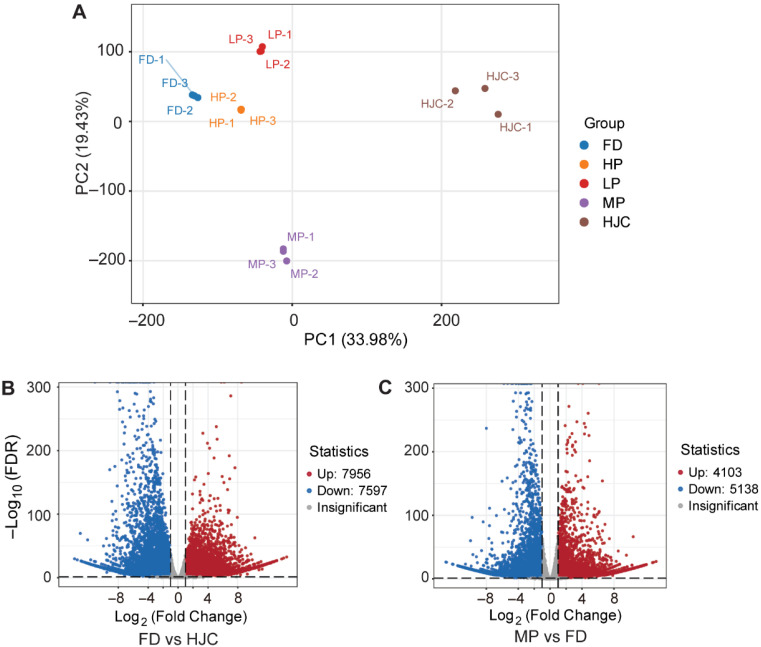
Differential gene expression in parental and hybrid tea samples. (**A**) Score plot of PCA for transcriptome datasets from the parental (FD, HJC) and F_1_ progeny (LP, MP and HP) tea samples. (**B**,**C**) Volcano plots of the gene expression differences between FD and HJC (**B**), and MP and FD samples (**C**).

To investigate differential gene expression between parental and offspring lines, we conducted principal component analysis (PCA) on the transcriptomic data of these samples. We found that the first component (PC1, R^2^ = 0.3398) separates the HJC and FD samples (Figure 1A), reflecting major differences in the gene expression levels between these two parent varieties. The second component (PC2, R^2^ = 0.1943) separates the MP and FD samples, also reflecting major differences in the gene expression levels between the F_1_ progeny and parental tea varieties. Thus, the observed PCA structure is mainly explained by genetic background, indicating that transcriptomic variation is largely genotype-dependent. We then conducted differential expression analysis among different sample groups, with a particular focus on HJC, FD, and MP. Between FD and HJC, 7956 genes showed significantly higher levels in FD, while 7597 genes showed lower levels (Figure 1B). Comparing MP with FD, 4103 genes showed significantly higher levels in MP, and 5138 genes showed lower levels (Figure 1C).

**Figure 2:**
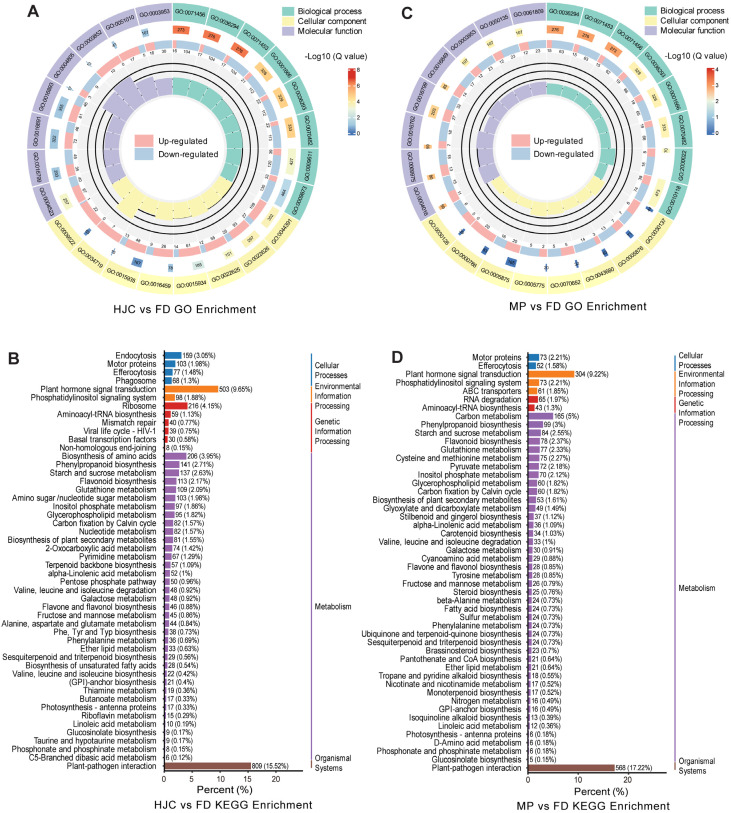
GO and KEGG enrichment analysis of differential expressed genes in different tea samples. (**A**,**C**) GO enrichment circle plot for the DEGs between HJC and FD (**A**), and MP and FD (**C**). This visualization depicts Gene Ontology (GO) enrichment results using concentric rings. The outermost ring shows the three major GO categories (Biological Process, Cellular Component, Molecular Function), color-coded by classification. The second ring displays the number of background genes per term and corresponding q-value, with bar length representing gene count and color intensity (gradated red) indicating enrichment significance. The third ring illustrates the proportion of up-regulated (pale red) and down-regulated (pale blue) genes, with specific values shown below. The innermost ring plots the Rich Factor (foreground/background gene count), with reference grid lines at 0.2 intervals. (**B**,**D**) KEGG enrichment barplot for the DEGs between HJC and FD (**A**), and MP and FD (**C**). The horizontal axis indicates the number of differentially expressed genes (DEGs) mapped to the pathway. The vertical axis lists the KEGG pathway names. Numeric labels within the plot represent the count of DEGs mapped to each pathway. Values in parentheses denote the ratio of DEGs mapped to the pathway versus the total number of annotated DEGs.

Gene Ontology (GO) enrichment analysis of differentially expressed genes between HJC and FD revealed significant enrichment in several biological processes, such as cellular response to hypoxia (GO:0071456), cellular response to decreased oxygen levels (GO:0036294), response to hypoxia (GO:0001666), response to wounding (GO:0009611), ethylene-activated signaling pathway (GO:0009873), and cellular response to ethylene stimulus (GO:0071369). In the molecular function category, significant enrichment was observed in RNA-DNA hybrid ribonuclease activity (GO:0004523), hydrolase activity for N-glycosyl compounds (GO:0016799), and trehalose-phosphatase activity (GO:0004805) (Figure 2A). KEGG pathway enrichment analysis showed significant enrichment in pathways including the phosphatidylinositol signaling system (ko04070) and plant hormone signal transduction (ko04075) belonging to signal transduction, and plant-pathogen interaction (ko04626) of environmental adaptation (Figure 2B). Additionally, pyrimidine metabolism (ko00240), nucleotide metabolism (ko01232), photosynthesis—antenna proteins (ko00196), inositol phosphate metabolism (ko00562), valine, leucine and isoleucine biosynthesis (ko00290), galactose metabolism (ko00052), terpenoid backbone biosynthesis (ko00900), and phosphonate and phosphinate metabolism (ko00440) were enriched (Figure 2B), supporting results from metabolomic pathway enrichment. These findings reveal substantial divergence between the two parental tea cultivars, HJC and FD, particularly in their responses to oxygen availability and the biosynthetic and metabolic pathways of primary metabolites.

GO enrichment analysis between the F_1_ progeny MP and parent sample FD showed significant differences in oxygen-related biological processes such as cellular response to oxygen levels (GO:0071453, GO:0036294, GO:0071456), response to jasmonic acid (GO:2000022), biosynthesis of green leaf volatiles (GO:0010597), responses to salicylic acid (GO:0009751), and glycerolipid metabolic processes (GO:0046486) (Figure 2C). In terms of molecular function, genes were significantly associated with adenylate cyclase activity (GO:0004016), cyclase activity (GO:0009975), hydrolyzing N-glycosyl compounds (GO:0016799), UDP-glucosyltransferase activity (GO:0035251), phosphorus-oxygen lyase activity (GO:0016849) and NAD+/NAD(P)+ nucleosidase activity (GO:0003953, GO:0050135, GO:0061809) (Figure 2C). KEGG analysis further revealed enrichment in linoleic acid metabolism (ko00591), carbon fixation in the Calvin cycle (ko00710), sesquiterpenoid and triterpenoid biosynthesis (ko00909), carotenoid biosynthesis (ko00906), phosphonate and phosphinate metabolism (ko00440), ABC transporters (ko02010), phosphatidylinositol signaling system (ko04070) and plant hormone signal transduction (ko04075) (Figure 2D). These results suggest substantial changes in both primary and secondary metabolic pathways as well as signaling and environmental response pathways in the F_1_ hybrid MP.

### 3.2. Metabolic Profiles of Tea

**Figure 3:**
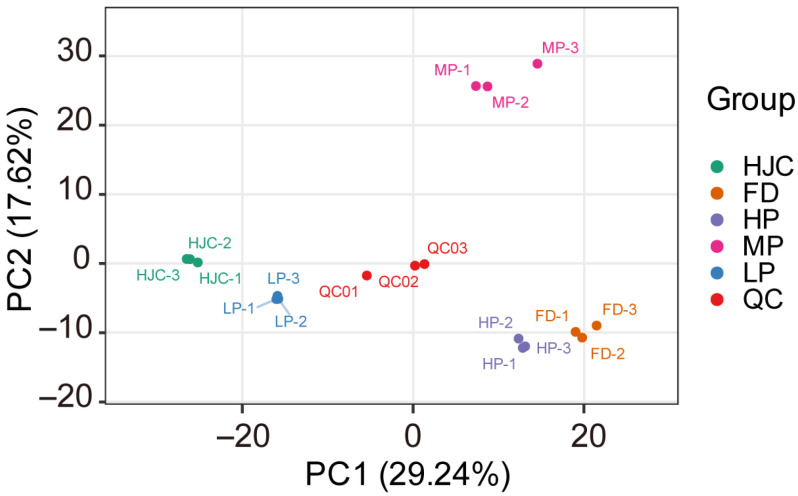
Score plot of PCA for metabolome datasets from the parental (FD, HJC) and F_1_ progeny (LP, MP and HP) tea samples. QC, quality control mix samples.

To investigate the metabolic changes in the various tea varieties, leaves from FD, HJC, HP, MP and LP, were also collected and then were used for the widely targeted metabolomics analysis based on LC-ESI-MS/MS. Among the results, we identified a total of 977 primary metabolite and its derivatives, including 338 amino acids, 128 nucleotide derivatives, 90 saccharides, 110 fatty acids, 134 organic acids, 45 glycerol esters and 24 vitamins. Principal component analysis (PCA) of the metabolite dataset showed that the first principal component (PC1, R^2^ = 0.292) distinguished the HJC and FD samples (Figure 3), indicating substantial differences in metabolite abundances between the two parental varieties. The second principal component (PC2, R^2^ = 0.176) separated the MP and FD samples, reflecting the major metabolic differences between the F_1_ progeny MP and the FD variety. Nevertheless, the relatively close clustering of HJC with LP and of FD with HP indicated smaller metabolic variations within these sample pairs.

**Figure 4:**
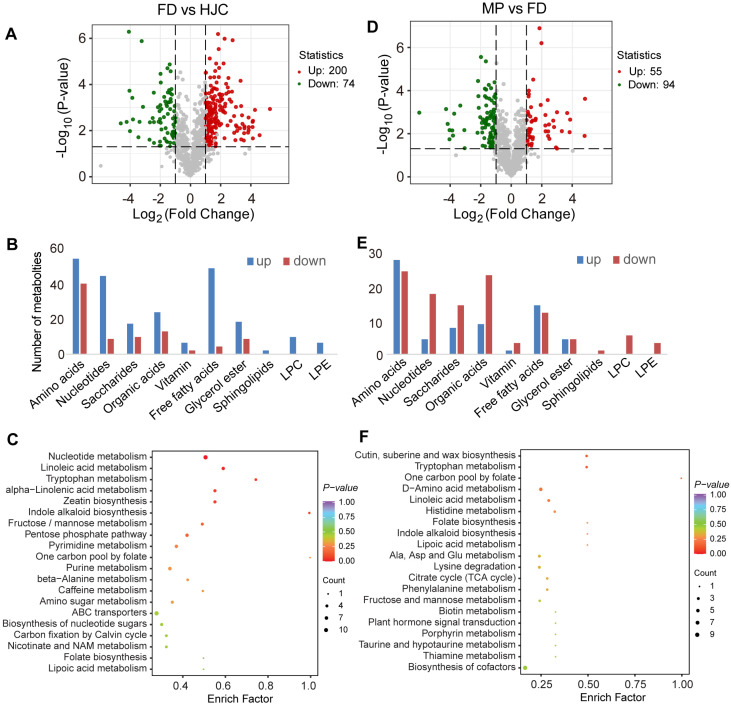
Differential metabolic accumulation in different tea samples. (**A**,**D**) Volcano plots of the metabolic differences between FD and HJC (**A**), and MP and FD samples (**D**). Each point represents a metabolite: green for significantly down-regulated, red for up-regulated, and gray for non-significant metabolites. The *x*-axis shows the log2-transformed fold change (Log2 FC) in relative abundance between two groups. Under the VIP + FC + *p*-value criteria, the *y*-axis represents significance level (−Log10 *p*-value), and dot size corresponds to VIP value. Under VIP + FC criteria, the *y*-axis denotes VIP value, with higher values indicating more reliable differential metabolites. (**B**,**E**) Numbers of differentially accumulated metabolites from different classes between FD and HJC (**B**), and MP and FD samples (**E**). (**C**,**F**) KEGG pathway enrichment analysis for the differentially accumulated metabolites between FD and HJC (**C**), and MP and FD samples (**F**).

To identify the most biologically meaningful metabolite changes, partial least squares–discriminant analysis (PLS-DA) was conducted. Variable importance in projection (VIP) scores ≥ 1.0 were used as criteria for selecting significant metabolites. Their metabolic alterations were visualized using volcano plots, in which red and green dots represent differential metabolites. Fold changes are displayed along the *X*-axis, and statistical significance (−log10 *p*-value) is shown on the *Y*-axis (Figure 4A). Between the FD and HJC varieties, VIP- and fold-change–based evaluation revealed significant differences in 294 metabolites (Q^2^ = 0.978 for PLS-DA). A total of 214 metabolites, including 50 amino acids and derivatives, 41 nucleotides and derivatives, 16 saccharides, 22 organic acids, 6 vitamin, 45 free fatty acids, 17 glycerol ester, 2 sphingolipids, 9 LPCs and 6 LPEs showed significantly higher levels in FD than that in HJC, while 80 metabolites, including 37 amino acids and derivatives, 8 nucleotides and derivatives, 9 saccharides, 12 organic acids, 2 vitamin, 4 free fatty acids and 8 glycerol ester, showed significant lower levels in FD than that in HJC (Figure 4B). KEGG pathway enrichment analysis of the differential metabolites revealed significant enrichment in nucleotide metabolism, linoleic acid metabolism, tryptophan metabolism, indole alkaloid biosynthesis, fructose and mannose metabolism, pentose phosphate pathway, pyrimidine metabolism, indicating substantial metabolic divergence between the FD and HJC varieties (Figure 4C). Moreover, the evaluation of the metabolite contents between MP and FD varieties revealed significant variations in 161 metabolites (Q^2^ = 0.954 for PLS-DA) (Figure 4D). A total of 62 metabolites, including 25 amino acids and derivatives, 4 nucleotides and derivatives, 7 saccharides, 8 organic acids, 1 vitamin, 13 free fatty acids and 4 glycerol ester, showed significantly higher levels in MP than that in FD, while 99 metabolites, including 22 amino acids and derivatives, 16 nucleotides and derivatives, 13 saccharides, 21 organic acids, 3 vitamin, 11 free fatty acids, 4 glycerol ester, 1 sphingolipids, 5 LPC and 3 LPE, showed significant lower levels in MP than that in FD (Figure 4E). Through KEGG pathway enrichment analysis of these differential metabolites, we observed significant enrichment in pathways of cutin, suberine and wax biosynthesis, tryptophan metabolism, one carbon pool by folate, amino acid metabolism, linoleic acid metabolism and histidine metabolism, revealing fundamental metabolic distinctions between MP and FD varieties (Figure 4F).

**Figure 5:**
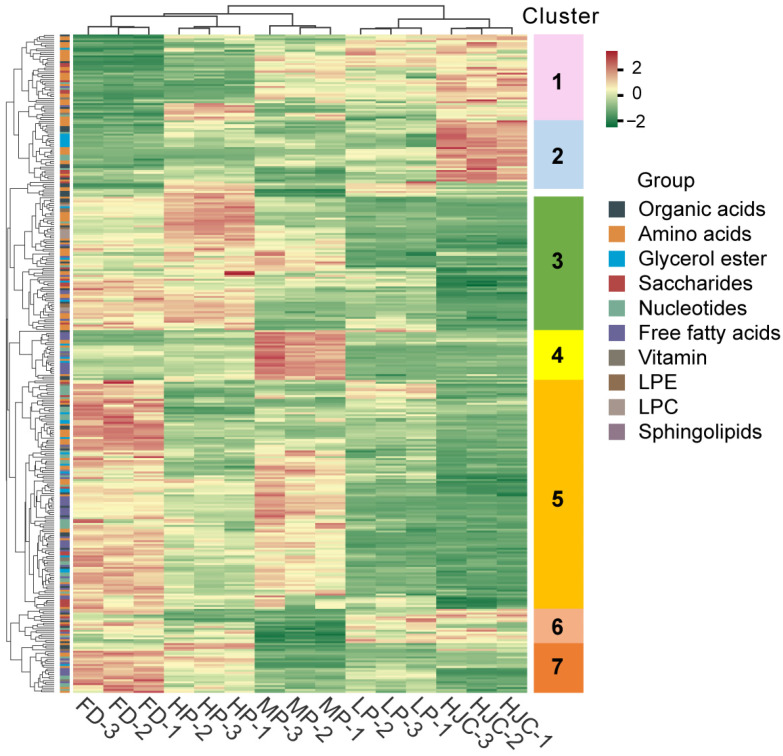
Heatmap visualization of relative differences in leaf metabolites that showed significant differential accumulation among different tea samples. The contents of each metabolite were normalized before performing linkage hierarchical clustering. Red indicates high abundance, and green indicates low relative metabolic content.

To characterize metabolite accumulation patterns across tea varieties, we performed hierarchical cluster analysis (HCA) of metabolites showing significant variation (Figure 5). The metabolites were grouped into seven major clusters, each reflecting distinct metabolic signatures among the parental lines (HJC and FD) and their F_1_ progeny. Clusters 1 and 2 were enriched for metabolites accumulating more strongly in HJC, including leucine derivatives, other amino acid derivatives, saccharides (cluster 1), and acylated tyrosine/tryptophan derivatives as well as several glycerol esters (cluster 2). In contrast, clusters 3, 5, and 7 were dominated by metabolites with higher abundances in FD, comprising diverse amino acid derivatives, nucleotides, free fatty acids, glycerol esters, and organic acids. These clusters capture the broad metabolic distinction between FD and HJC and include many metabolite classes later shown to contribute strongly to metabolic heterosis. Importantly, cluster 4 represents the most biologically relevant pattern in the context of hybrid vigor. Although fewer in number, metabolites in this cluster—primarily free fatty acids and a small set of nucleotide derivatives—show their highest levels in the F_1_ progeny MP, exceeding both parents. This pattern highlights a subset of metabolites exhibiting pronounced hybrid enhancement.

### 3.3. Heterosis Level of Metabolites in the Hybrids

**Figure 6:**
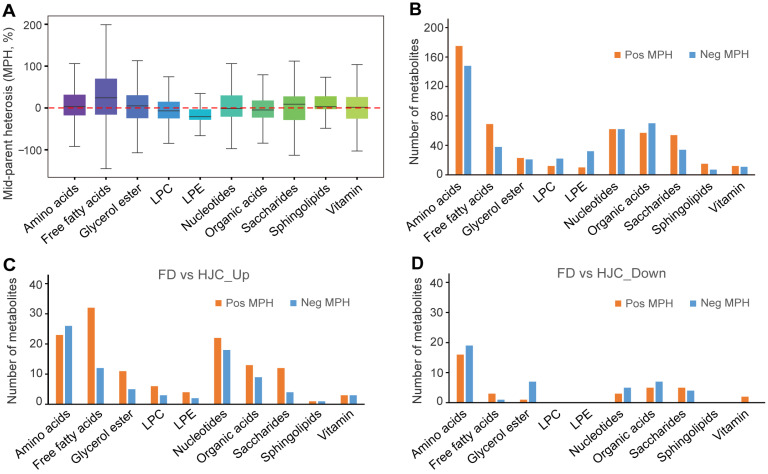
Statistical analysis of the metabolites that showed heterosis in tea samples. (**A**) Boxplot showing the distribution of relative MPH in each metabolic category in F_1_ progeny MP sample. (**B**) numbers of both positive and negative MPH metabolites from different classes in MP sample. (**C**,**D**) Numbers of differential metabolites between FD and HJC that showed both positive and negative MPH metabolites from different classes in MP.

To better understand the degree of tea heterosis in F_1_ progeny, we next investigated how many of the metabolites in the hybrids show non additive patterns of accumulation deviating from the mid-parent value (MPV). Among the 977 metabolites identified/annotated, 489 (50.05%) were identified as non-additively accumulated in the F_1_ progeny MP comparing to its parental lines (Figure 6A). Moreover, for the metabolites in different class, 175 amino acid and its derivatives, 69 free fatty acids, 15 sphingolipids, 54 saccharides showed positive MPV. In contrast, 148 amino acid and its derivatives, 38 free fatty acids, 7 sphingolipids, 34 saccharides showed negative MPV (Figure 6B), indicating significant metabolic heterosis of these metabolites in the HJC and FD hybrids. Further analysis of metabolites significantly differing between FD and HJC revealed distinct MPV distributions. Among the 214 metabolites significantly elevated in FD, 127 exhibited positive MPV while 83 exhibited negative MPV (Figure 6C). Conversely, of the 80 metabolites significantly elevated in HJC, 35 displayed positive MPV and 45 displayed negative MPV (Figure 6D). Notably, within the metabolites enriched in FD (Female parent), majority of the free fatty acids (32/44), glycerol esters (11/16, mainly represented by the glycosylated forms), and saccharides (12/16) presented positive MPV (Figure 6C). Within the metabolites enriched in HJC (Male parent), however, we observed that majority of the glycerol esters which were mainly represented by linolenoyl-glycerol, showed positive MPV (Figure 6D). These findings suggested that these three metabolite classes are more likely to exhibit hybrid dominance in crosses.

### 3.4. Analysis of Pathways Related to Tea

**Figure 7:**
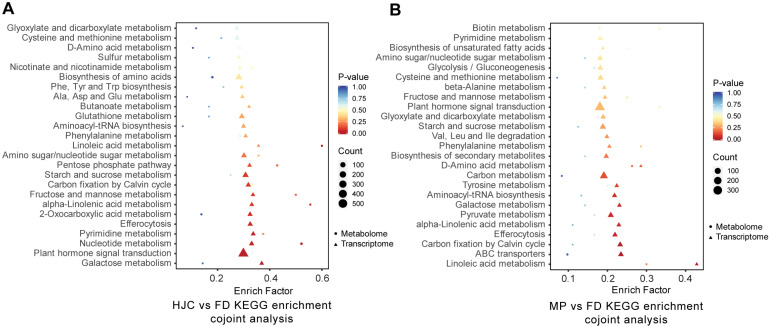
Metabolomics and transcriptomics co-joint analysis for both differential metabolites and genes among different tea samples. Bubble plot of KEGG enrichment analysis for both differential metabolites and genes between HJD and FD (**A**), and MP and FD tea samples (**B**).

To elucidate changes in metabolic pathways and pinpoint key pathways and genes contributing to metabolic variation across tea varieties and hybrids, we performed integrated transcriptomic and metabolomic analyses. In comparing HJC and FD, both transcriptomic and metabolomic KEGG enrichment showed significant differences in pathways such as galactose metabolism (ko00052), plant hormone signal transduction (ko04075), nucleotide metabolism (ko01232), and pyrimidine metabolism (ko00240) (Figure 7A). These pathways exhibited not only gene expression differences but also differential accumulation of corresponding metabolites. Between MP and FD, we detected significant differences in gene expression and metabolite accumulation in linoleic acid metabolism (ko00591), ABC transporters (ko02010), carbon fixation (ko00710), efferocytosis (ko04148), alpha-linolenic acid metabolism (ko00592), pyruvate metabolism (ko00620), and galactose metabolism (ko00052) (Figure 7B).

**Figure 8:**
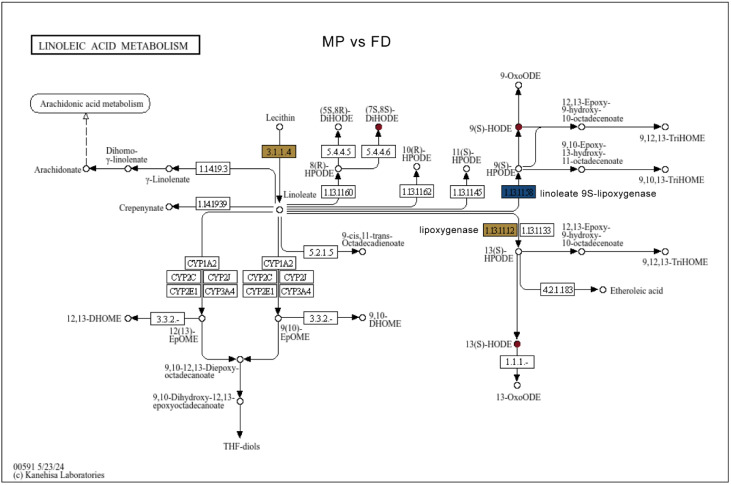
Linoleic acid metabolism pathway enriched for differential metabolites and genes between MP and FD. Red indicates up-regulated genes or metabolites, blue indicates down-regulated genes or metabolites, and yellow indicates genes or metabolites containing both up- and down-regulated states.

In linoleic acid metabolism, metabolites including (7S,8S)-DiHODE, (9Z,11E)-(13S)-13-Hydroperoxyoctadeca-9,11-dienoic acid, 13(S)-HODE, 9(S)-HODE, 9(S)-HPODE, 9,10,13-TriHOME and 9,12,13-TriHOME were significantly more abundant in FD, with MP showing the highest levels, reflecting hybrid vigor. Lipoxygenase (EC: 1.13.11.12) genes responsible for synthesizing 13(S)-HPODE, 13(S)-HODE and 9,12,13-TriHOME compounds, such as *TEA003727*, *TEA011765*, and *novel.6290*, exhibited markedly elevated expression in the F_1_ hybrid MP relative to both parental lines, FD and HJC (Figure 8). To further validate the gene expression, we performed quantitative PCR (qPCR). The results showed that the expression levels of *TEA003727* and *TEA011765* were highest in MP among the five tea cultivars, and were significantly higher than those in FD, with increases of 2.04-fold and 3.11-fold, respectively (Table 2). We subsequently performed correlation analysis for key gene–metabolite pairs. Within the two-group comparison, 13(S)-HODE showed strong positive correlations with *TEA003727* and *TEA011765*, with r-values of 0.955 and 0.960, respectively. However, when the analysis was expanded to all five genotypes, the correlations between 13(S)-HODE and these genes decreased markedly (r = −0.42, −0.38, and −0.65), likely reflecting the greater variability in gene expression and metabolite accumulation across a wider set of cultivars.

Metabolites such as 9(S)-HPODE, 9(S)-HODE, 9,10,13-TriHOME, and 9,12,13-TriHOME also exhibited similar accumulation patterns, with significantly higher levels in MP compared to other tea varieties (Figure 8). Furthermore, these metabolites in FD were significantly higher than those in HJC, with respective increases of 3.17-fold, 2.37-fold, 3.54-fold, and 3.83-fold. The gene *TEA025499*, which encodes linoleate 9S-lipoxygenase (EC: 1.13.11.58) responsible for the biosynthesis of these compounds, was expressed at a significantly higher level in FD than in HJC—approximately 3.47-fold greater—but displayed lower expression in MP. Our qPCR results validated the transcriptomic data, showing that the expression of this gene in FD was 6.73-fold that in HJC and 5.26-fold that in MP. These findings suggest that other genes may also be involved in the regulation of this pathway (Table 2). In contrast, another linoleate 9S-lipoxygenase gene, *TEA015727*, was also significantly upregulated in FD relative to HJC by approximately 3.47-fold. Notably, *TEA015727* showed the highest expression in MP, with transcript levels 1.71 times higher than in FD and 5.94 times higher than in HJC. This finding is consistent with the qPCR results, which indicated that the gene expression was highest in MP, and that the expression level in FD was 3.31-fold higher than that in HJC. These findings suggest that the F_1_ hybrid MP exhibits strong coordination between metabolite accumulation and gene expression within the linoleic acid metabolic pathway. The correlation between 9(S)-HODE and *TEA015727* reached an r-value of 0.963, indicating a highly consistent accumulation pattern between this metabolite and the gene. Overall, MP inherited the high metabolic activity of FD, with most linoleic acid-derived metabolites present at levels comparable to or exceeding those in FD, and the expression patterns of key enzymatic genes remained consistent with those of the maternal line, indicating a synchronously regulated metabolic-transcriptional network.

**Figure 9:**
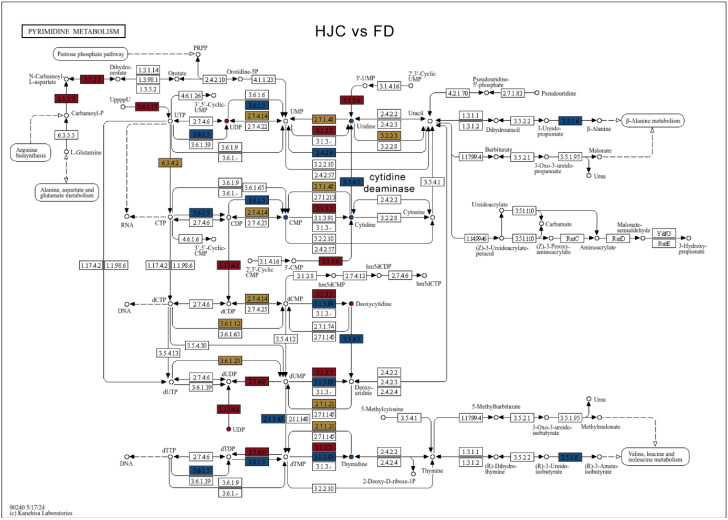
Pyrimidine metabolism pathway enriched for differential metabolites and genes between HJC and FD. Small circles represent metabolites and squares represent genes. Red indicates up-regulated genes or metabolites, blue indicates down-regulated genes or metabolites, and yellow indicates genes or metabolites containing both up- and down-regulated states.

Based on these findings, pyrimidine metabolism and linoleic acid metabolism appear to undergo pronounced changes during hybridization and contribute to metabolic heterosis in tea. In the pyrimidine metabolism pathway, HJC showed lower uridine content than FD, while expression of cytidine deaminase (EC: 3.5.4.5; *TEA005242*), which converts cytidine to uridine, was significantly lower in HJC than that in FD (Figure 9). The gene expression level of *TEA005242* was further confirmed by quantitative PCR, showing a 4.3-fold decrease in HJC (Table 2). In the two-group comparison, uridine and *TEA005242* showed a moderate positive correlation (r = 0.78). The CMP levels were lower in HJC, likely due to decreased expression of uridine kinase genes (EC: 2.7.1.48), including *TEA007128* and several novel genes such as *novel.17005*, *novel.3896*, *novel.5114*, and *novel.8127*. Cytidine and *TEA007128* exhibited a strong negative correlation (r = –0.84), consistent with the reduced nucleotide phosphorylation capacity in HJC. Reduced UDP and thymidine levels in HJC further corresponded to lower expression of apyrase and the 5′-deoxynucleotidase (EC: 3.1.3.5) gene *novel.22032*.

## 4. Discussion

Non-additive gene expression patterns are central to heterosis in tea hybrids, as evidenced by transcriptomic data where 38–41% of genes display non-additive expression, in contrast to a minor fraction (7.83%) exhibiting additive behavior [11,12]. This dominance is characterized by high parental and over-dominance expression modes, with KEGG and GO analyses confirming enrichment in pathways such as plant hormone signal transduction, biological regulation, and metabolic processes [11,12]. These patterns suggest that the hybrid vigor arises from the synergy of multiple genetic interactions rather than simple additive inheritance. Supporting this, functional annotations indicate that non-additive genes regulate critical cellular processes, including photosynthesis and respiration, which enhance overall plant performance [12,31]. Thus, the predominance of non-additive expression underscores its fundamental role in conferring heterosis, aligning with models that emphasize the composite nature of hybrid superiority over the parental average [12].

Metabolomic heterogeneity in tea hybrids manifests primarily as non-additive patterns in metabolite accumulation, where nearly 10% of metabolites demonstrate effects such as over-dominance, under-dominance, and high-parent dominance [16,32,33]. Notably, the observed ~50% rate of non-additive metabolite accumulation in our tea F_1_ hybrids is considerably higher. This discrepancy likely stems from two key factors. First, the parental cultivars HJC and FD exhibit pronounced metabolic divergence, with nearly 300 significantly different metabolites, increasing the probability of dominant or overdominant inheritance patterns. Second, the use of a widely targeted LC–MS/MS platform in our study enabled the detection of a number metabolites, providing broader chemical coverage and greater sensitivity than previous methods and thus capturing more subtle differences between hybrids and parents. This is further contrasted with the predominantly additive metabolite profiles observed in the majority of compounds [32]. Significantly, metabolites involved in photosynthetic pathways show positive mid-parent heterosis (MPH), while those in photorespiratory pathways exhibit negative MPH [16], suggesting that hybrids optimize carbon assimilation efficiency through balanced metabolic regulation. The developmental dependence of metabolite composition further indicates that genotypic differences are often masked by kernel age or environmental factors [14,32,34], reinforcing the dynamic nature of metabolic heterosis. Robust metabolite levels in hybrids correlate with biomass variance (explaining up to 37–44% in roots and leaves), highlighting a trade-off between defense-related metabolites and performance traits [14]. Collectively, these findings reveal that metabolic heterosis is relatively mild compared to trait heterosis, with non-additive metabolite shifts supporting enhanced physiological resilience [16].

A notable outcome of our study is the clear enrichment of linoleic acid metabolism in F_1_ hybrids, reflected in both elevated metabolite levels and increased expression of pathway-related genes. We demonstrate that linoleic acid metabolism, a pathway not previously emphasized in Camellia heterosis studies, plays a pivotal role in the metabolic reprogramming of F_1_ tea hybrids. We observed markedly increased levels of the oxylipins 9(S)-HODE and 13(S)-HODE in the hybrids, together with higher expression of lipoxygenase genes such as *TEA011765* and *TEA015727*. This transcription–metabolite concordance suggests a tightly regulated lipid remodeling mechanism underpinning hybrid vigor. We also detected coordinated changes in pyrimidine metabolites and the expression of cytidine deaminase and uridine kinase genes, suggesting a nucleotide remodeling process not previously reported in Camellia heterosis [4,10]. Our findings provide new insights that F_1_ tea hybrids engage distinct, pathway-specific transcriptional–metabolic integration, highlighting the importance of linoleic acid and pyrimidine metabolism in perennial heterosis beyond previously documented volatile or catechin-related traits. While this study emphasized primary metabolic pathways, further exploration of secondary metabolites will be valuable for understanding additional layers of regulatory complexity underlying hybrid vigor.

The elevated oxylipin levels in the hybrids suggest enhanced signaling capacity, as these molecules participate in regulating growth, senescence, and stress responses [35,36,37]. Consistent with these metabolite patterns, LOX and HPL transcripts were also elevated in the hybrids. Similar lipid-related transcriptional activation was observed in maize hybrids, where temporal regulation of LOX genes contributed to enhanced carbon assimilation and biomass production [16]. Together, these results indicate that lipid remodeling contributes to heterosis through coordinated transcriptional and metabolic changes. These combined metabolite and gene expression shifts point toward an enhanced oxylipin signaling landscape in the hybrids. Oxylipins act as potent signaling molecules in plants, modulating phytohormone pathways and stress-response networks. For example, jasmonate-family oxylipins produced via the 13-LOX branch (through the allene oxide synthase pathway) orchestrate defense gene activation and also influence developmental processes. In parallel, 9-LOX pathway products can engage redox-regulatory networks and contribute to induced systemic resistance. Unlike the frequently reported negative heterosis of catechins in tea hybrids, our study revealed clear positive heterosis for amino acids and lipids, with metabolites such as proline, leucine, and tryptophan derivatives highly enriched in the F_1_ genotype MP [38,39,40]. This pattern is consistent with findings in other crops showing that enhanced amino acid biosynthesis contributes to biomass heterosis. In contrast, secondary metabolites like catechins and flavonoids often decrease in hybrids, reflecting downregulation of phenylpropanoid- and flavonoid-related pathways [41,42]. These contrasting trends highlight the metabolite-specific nature of heterosis and identify amino acids and lipids as promising targets for improving hybrid vigor and quality.

In tea, F_1_ hybrids exhibit extensive non-additive gene expression and metabolite accumulation, notably elevated linoleic acid derivatives and nucleotides alongside upregulation of fatty acid and nucleotide metabolism genes based on our study. This coordinated transcript–metabolite shift suggests a transcriptionally driven remodeling of lipid pathways contributing to hybrid vigor. In perennial crops, integrative omics approaches have begun to reveal molecular heterosis mechanisms that parallel those in annuals. In citrus, an integrative study of mandarin–orange tangor hybrids found pronounced fruit metabolome shifts: sugars and vitamin C increased while organic acids declined during ripening, reflecting enhanced sweetness and fruit quality in the hybrid [43]. Poplar tree hybrids likewise show growth heterosis associated with non-additive expression of ~44 genes in photosynthetic carbon fixation, starch/sucrose turnover, and nitrogen metabolism; collectively these genes boost carbon fixation and nitrogen use efficiency while reducing respiratory costs [44]. Notably, such patterns echo those in rice and maize, where hybrids demonstrate enhanced photosynthesis and nitrogen utilization along with elevated nucleotide and amino acid metabolism linked to superior biomass and yield. Taken together, these findings underscore the promise of integrative transcriptomic–metabolomic approaches to decipher heterosis in long-lived species [45].

The integrated approach combining transcriptomics and metabolomics provides a robust framework for understanding tea hybrid heterosis, with implications for accelerating genetic breeding [2,5,46,47]. Omics-based strategies, such as those detailed in Camellia transcriptome analyses, enable comprehensive gene expression profiling across developmental stages and stresses, facilitating the identification of metabolic pathways and candidate genes [2,48]. These resources support the development of predictive models where metabolites serve as proxies for integrated genetic networks, as hypothesized for yield traits [14,22]. Future research should focus on leveraging non-additive mechanisms for the targeted breeding of high-volatile or anthocyanin-rich cultivars, using insights from enriched pathways like oxidative phosphorylation and plant hormone signaling [11,38,49,50]. Limitations include the masking effect of developmental dynamics on genotypic differences [32,51], necessitating longitudinal studies.

## 5. Conclusions

This study provides new insights that hybrid vigor in tea (*C. sinensis*) arises from coordinated non-additive patterns at both transcriptomic and metabolomic levels. F_1_ hybrids derived from HJC and FD exhibited widespread non-additive accumulation of metabolites, particularly within lipid and nucleotide categories, and upregulated expression of key genes involved in linoleic acid metabolism, pyrimidine biosynthesis, and stress response. Notably, the strong enrichment of linoleic acid derivatives such as 9(S)-HODE and 13(S)-HODE, alongside the upregulation of lipoxygenase-related genes, suggests that the transcriptionally driven remodeling of lipid pathways contributes substantially to metabolic heterosis. Integrative pathway analyses further highlighted tight gene–metabolite co-regulation, particularly within the linoleic acid and pyrimidine metabolism pathways. These findings underscore the complexity of tea hybrid heterosis, where both primary and specialized metabolites play synergistic roles. Moreover, this study demonstrates the utility of combining RNA-seq and widely targeted metabolomics to uncover multi-layered regulatory networks underlying trait enhancement in perennial crops. The identification of specific metabolites and gene targets associated with hybrid advantage offers valuable molecular markers for future breeding programs aiming to develop elite tea cultivars with superior metabolic profiles and improved agronomic performance. Key lipoxygenase genes and their associated linoleic acid derivatives may serve as reliable candidate markers for selecting high-performing hybrids. These targets can be incorporated into molecular breeding pipelines to enable early-stage screening and the pyramiding of favorable alleles. The integration of metabolite-based markers with transcriptomic indicators may enhance the precision and efficiency of marker-assisted selection in *C. sinensis* breeding programs. Further investigations across developmental stages and environmental contexts are warranted to refine predictive models of heterosis and to fully harness the genetic potential of tea hybrids.

## Figures and Tables

**Table 1:** Sequencing data statistics derived from the five tea varieties.

Sample	Raw Reads	Clean Reads	Clean Base (G)	Q20 (%)	Q30 (%)	GC Content (%)
FD-1	63,718,424	58,939,750	8.84	98.55	95.67	44.14
FD-2	62,962,102	59,128,246	8.87	98.5	95.51	44.58
FD-3	59,583,264	54,718,768	8.21	98.57	95.76	43.91
HJC-1	56,206,406	54,353,600	8.15	98.55	95.12	43.38
HJC-2	43,807,728	42,219,234	6.33	98.63	95.57	43.84
HJC-3	50,913,026	49,370,598	7.41	98.26	94.29	43.53
HP-1	72,515,996	66,511,350	9.98	98.55	95.71	45.68
HP-2	75,290,642	68,759,084	10.31	98.55	95.65	45.36
HP-3	55,348,690	49,350,924	7.4	98.55	95.7	44.24
LP-1	61,701,412	56,520,842	8.48	98.57	95.72	44.23
LP-2	65,546,398	60,254,832	9.04	98.58	95.77	45.17
LP-3	71,450,892	65,138,682	9.77	98.57	95.71	45.27
MP-1	67,535,352	62,258,770	9.34	98.59	95.8	44.66
MP-2	51,862,584	47,020,250	7.05	98.59	95.8	43.67
MP-3	59,940,430	56,224,966	8.43	98.58	95.72	44.46
Total	918,383,346	850,769,896	127.61			

**Table 2:** Candidate gene expression in the five tea cultivars validated by qPCR.

	Relative Gene Expression					FD/HJC		MP/FD	
Gene	HJC	FD	HP	LP	MP	*p* Value	Fold Change	*p* Value	Fold Change
TEA003727	1.35 × 10^−1^	1.57 × 10^−1^	2.57 × 10^−1^	1.38 × 10^−1^	3.19 × 10^−1^	1.50 × 10^−2^	1.16	2.3 × 10^−3^	2.04
TEA011765	4.80 × 10^−1^	4.28 × 10^−1^	7.06 × 10^−1^	3.35 × 10^−1^	1.33	1.22 × 10^−1^	0.89	5.4 × 10^−6^	3.11
TEA025499	7.69 × 10^−3^	5.17 × 10^−2^	5.42 × 10^−2^	2.55 × 10^−2^	9.83 × 10^−3^	7.09 × 10^−4^	6.73	8.7 × 10^−4^	0.19
TEA015727	3.27 × 10^−2^	1.08 × 10^−1^	1.04 × 10^−1^	5.22 × 10^−2^	1.48 × 10^−1^	3.64 × 10^−3^	3.31	4.9 × 10^−3^	1.37
TEA005242	4.64 × 10^−3^	2.03 × 10^−2^	1.65 × 10^−2^	8.07 × 10^−3^	1.47 × 10^−2^	3.20 × 10^−3^	4.37	3.2 × 10^−2^	0.72

## Data Availability

The sequence data of the 15 tea samples have been deposited in NCBI under the BioProject accession number PRJNA1268759. The original contributions presented in this study are included in the article. Further inquiries can be directed to the corresponding author.
